# Occult gastric carcinoma with microsatellite instability diagnosed 10 years after excision of metastatic lymph node: a case report

**DOI:** 10.1186/s40792-024-01988-6

**Published:** 2024-08-09

**Authors:** Yutaka Tamamori, Takuya Mori, Akihiro Tanaka, Takuma Okada, Shogo Tanaka, Yuichi Fumimoto, Kiyotaka Yukimoto, Ryugo Sawada, Hisao Sano, Yoshio Ohta, Hirokazu Taniguchi, Toshimasa Tsujinaka

**Affiliations:** 1 Department of Gastroenterological Surgery, Izumi City General Hospital, 4-5-1 Wake-Cho, Izumi, Osaka 594-0073 Japan; 2 Department of Hepato-Biliary-Pancreatic Surgery, Izumi City General Hospital, Osaka, Japan; 3 Department of Pathology, Kameda Medical Center, Chiba, Japan; 4 Department of Diagnostic Pathology, Izumi City General Hospital, Osaka, Japan; 5 Department of Pathology, Faculty of Medicine, Kyorin University, Tokyo, Japan

**Keywords:** Gastric carcinoma, Occult, Microsatellite instability

## Abstract

**Background:**

Suprapancreatic lymph node metastasis is one of the usual routes for gastric cancer. However, it is rare for the primary lesion to be found several years after resection of the suprapancreatic metastatic lymph node. This is a report of occult gastric carcinoma with microsatellite instability diagnosed 10 years after excision of a metastatic lymph node.

**Case presentation:**

A 55-year-old female presented with suprapancreatic lymph node swelling during a medical examination. Gastroscopy revealed no malignancy. We performed an excisional biopsy via laparotomy and histologically suspected metastatic cancer of unknown origin. After nine and a half years, we detected early gastric cancer by gastroscopy and performed a distal gastrectomy. The gastric tumor was pathologically similar to the previous suprapancreatic tumor. Immunohistochemical examination revealed that both the stomach and suprapancreatic lymph node exhibited microsatellite instability, suggesting that the two lesions were of the same origin.

**Conclusions:**

This case is considered valuable because there have been no previous reports of gastric cancer with characteristics of high microsatellite instability in which the primary tumor was identified a long time after resection of metastatic lesions.

## Background

Gastric cancer (GC) was responsible for over one million new cases in 2020, with 769,000 estimated deaths, ranking fifth in incidence and fourth in mortality globally [1]. GC is one of the most common cancers in the East Asian population. GC most commonly metastasizes to the lymph nodes in both the perigastric and extraperigastric regions, and suprapancreatic lymph node metastasis (LNM) is particularly prevalent. Typically, when the suprapancreatic lymph nodes are enlarged, metastasis from gastric, pancreatic, or biliary tract cancer is suspected first. We encountered a case in which a large lymph node with suspected metastasis was found at the suprapancreatic margin; however, a primary diagnosis was not made at the time. Ten years after lymph node removal, the patient underwent gastrectomy for gastric carcinoma with microsatellite instability (MSI). In this report, we examine the relationship between these two lesions, which are histologically similar, although the resection dates were 10 years apart.

## Case presentation

**Fig. 1:**
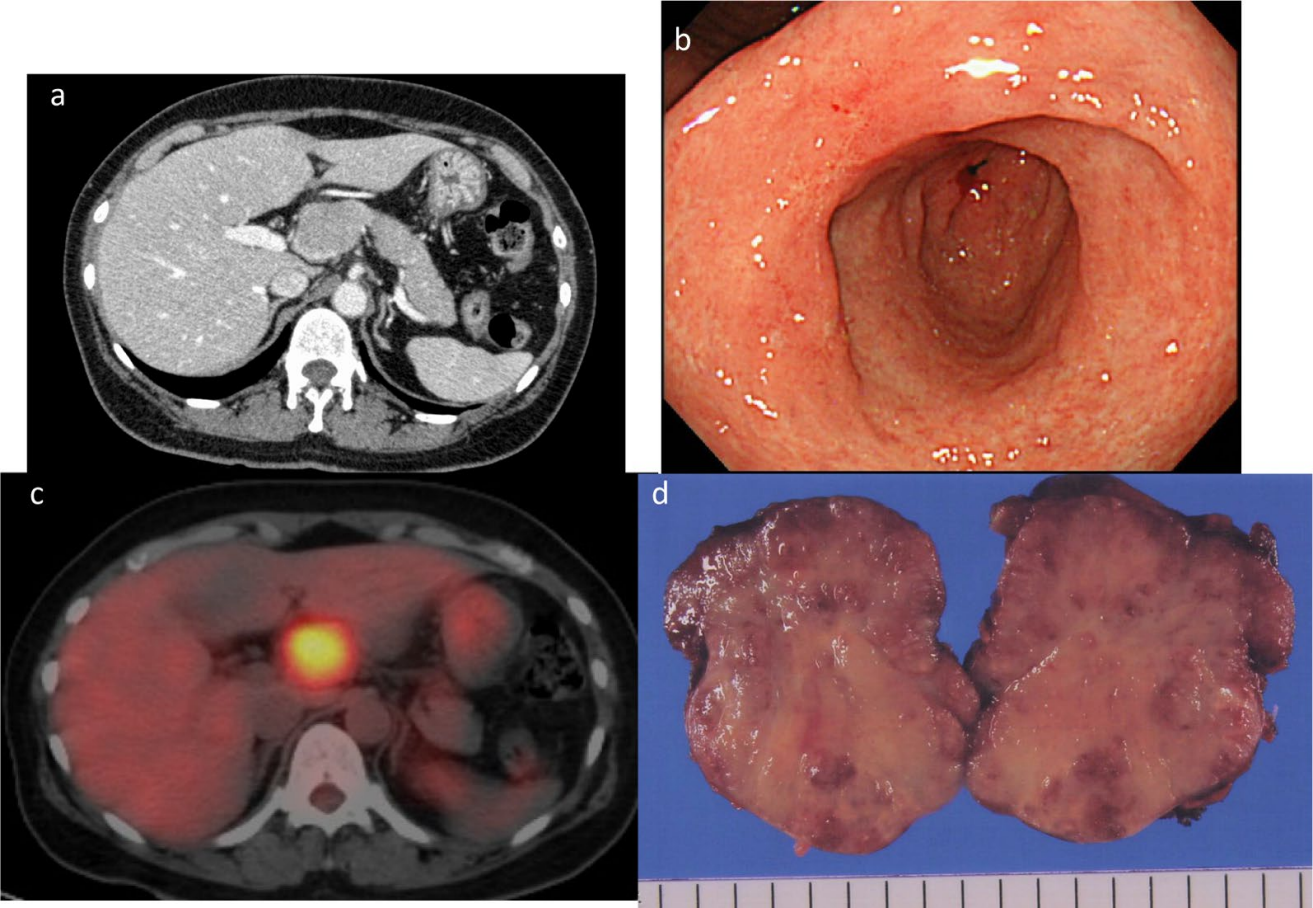
Images before lymphadenectomy. **a** CECT revealed the existence of a relatively uniformly enhanced nodule in contact with the upper edge of the pancreatic head. **b** Gastroscopy showed a small ulcer scar at the angle of stomach. **c** PET showed the accumulation of F-18-FDG only in the suprapancreatic nodule, as demonstrated by CECT (maximum standardized uptake value: 5.83). **d** Macroscopic findings from the resected lymph node showed solid, elastic, soft, and proliferation in an expansive manner (size: 51 × 42 × 31 mm). *CECT* contrast-enhanced abdominal computed tomography, *F-18-FDG* fluorine-18-fluorodeoxyglucose, *PET* positron emission tomography

**Fig. 2:**
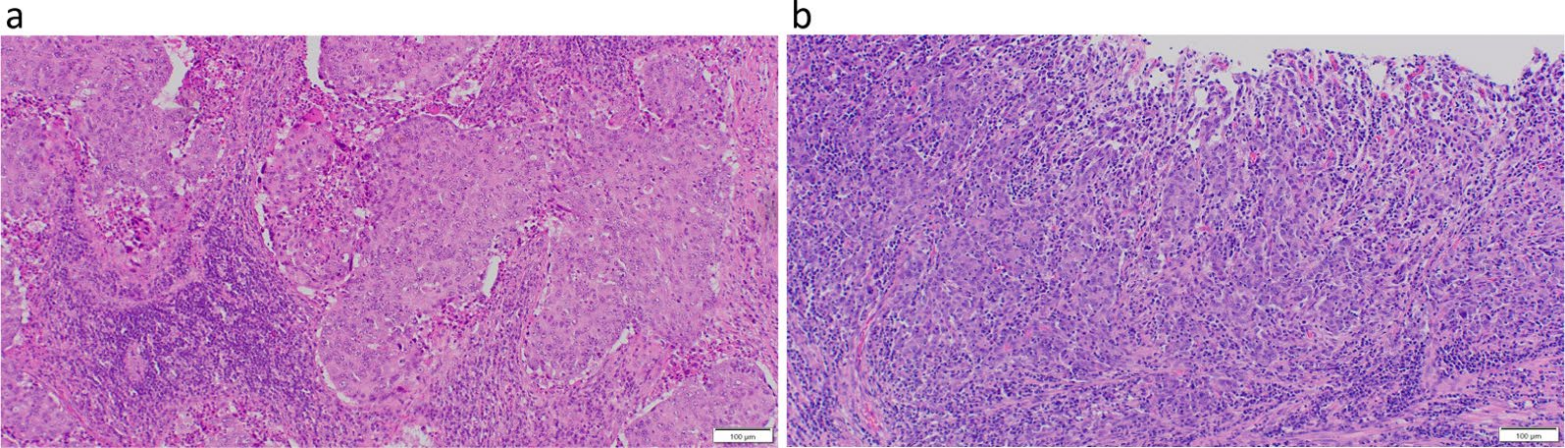
Histopathological findings. **a** Suprapancreatic lymph node (× 100). Malignant cells were infiltrative and proliferative, forming irregular foci of various sizes in the lymph node. **b** Stomach lesion (× 100). Clusters of atypical cells proliferated from the alveolar to the solid layers. Individual cells showed enlarged nuclei with polygonal and clear nucleoli. Lymphocytes were densely infiltrated around the tumor cells, and lymphoid follicles formed in the periphery

A 55-year-old female presented with an intra-abdominal mass detected during a medical checkup. The patient had no complaints related to the mass. Contrast-enhanced abdominal computed tomography (CECT) revealed a relatively uniformly enhanced nodule in contact with the upper edge of the pancreatic head (Fig. 1a). Although gastroscopy indicated a small ulcer scar at the angle of the stomach, biopsy specimens revealed no evidence of malignancy (Fig. 1b). No searches were made for *Helicobacter pylori (H. pylori)*. Positron emission tomography (PET) showed accumulation of fluorine-18-fluorodeoxyglucose (F-18-FDG) only in the suprapancreatic nodule, as indicated by CECT with a maximum standardized uptake value of 5.83 (Fig. 1c). The laboratory analyses revealed no abnormalities. Tumor biomarkers (carcinoembryonic antigen, carbohydrate antigen [CA]19-9, CA 125, squamous cell carcinoma antigen, neuron-specific enolase, and soluble interleukin-2 receptor) were all within the normal ranges. The patient was clinically diagnosed with LNM from an unknown primary origin or a primary lymph node tumor (e.g., malignant lymphoma). To obtain the histological diagnosis, we performed an excisional biopsy via laparotomy. The tumor was in contact with the common hepatic artery; however, as there was no invasion, we resected the tumor without injuring the artery. The tumor was solid, elastic, soft, and expansively proliferated, with a size of 51 × 42 × 31 mm (Fig. 1d). Histological examination revealed that the malignant cells were infiltrating and proliferating, forming irregular foci of various sizes within the lymph node (Fig. 2a). We suspected a metastatic tumor of unknown origin.

**Fig. 3:**
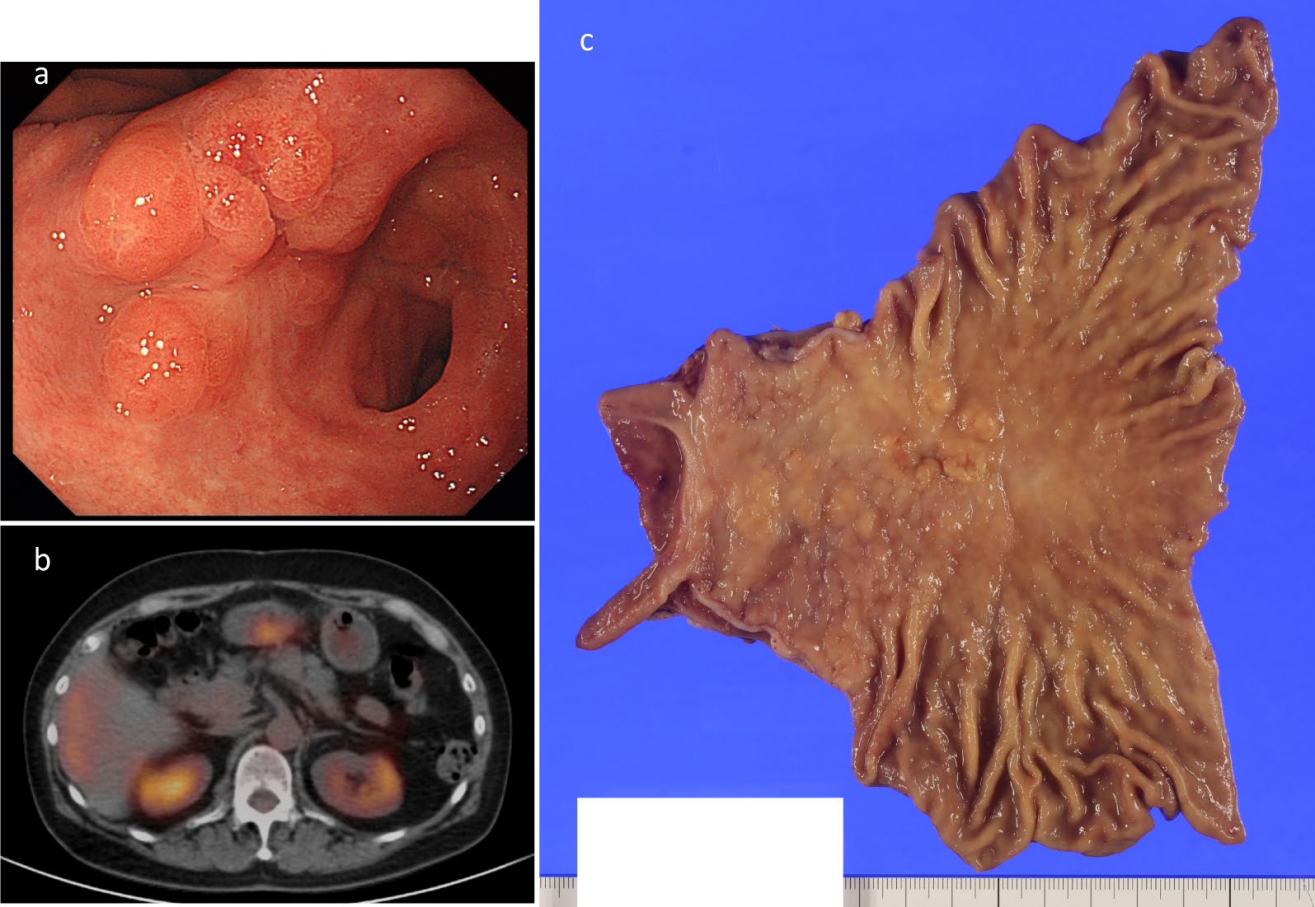
Images before gastrectomy. **a** Gastroscopy showed a mixed raised and depressed lesion (0-IIa + IIc) at the angle of the previously identified ulcer scar. **b** PET showed the accumulation of F-18-FDG only in the main lesion on the stomach. **c** On macroscopic examination of the resected specimen, a 0-IIa + IIc tumor that was 70 × 50 mm in size was seen extending from the lesser curvature to the posterior wall of the lower body. *F-18-FDG* fluorine-18-fluorodeoxyglucose, *PET* positron emission tomography

**Fig. 4:**
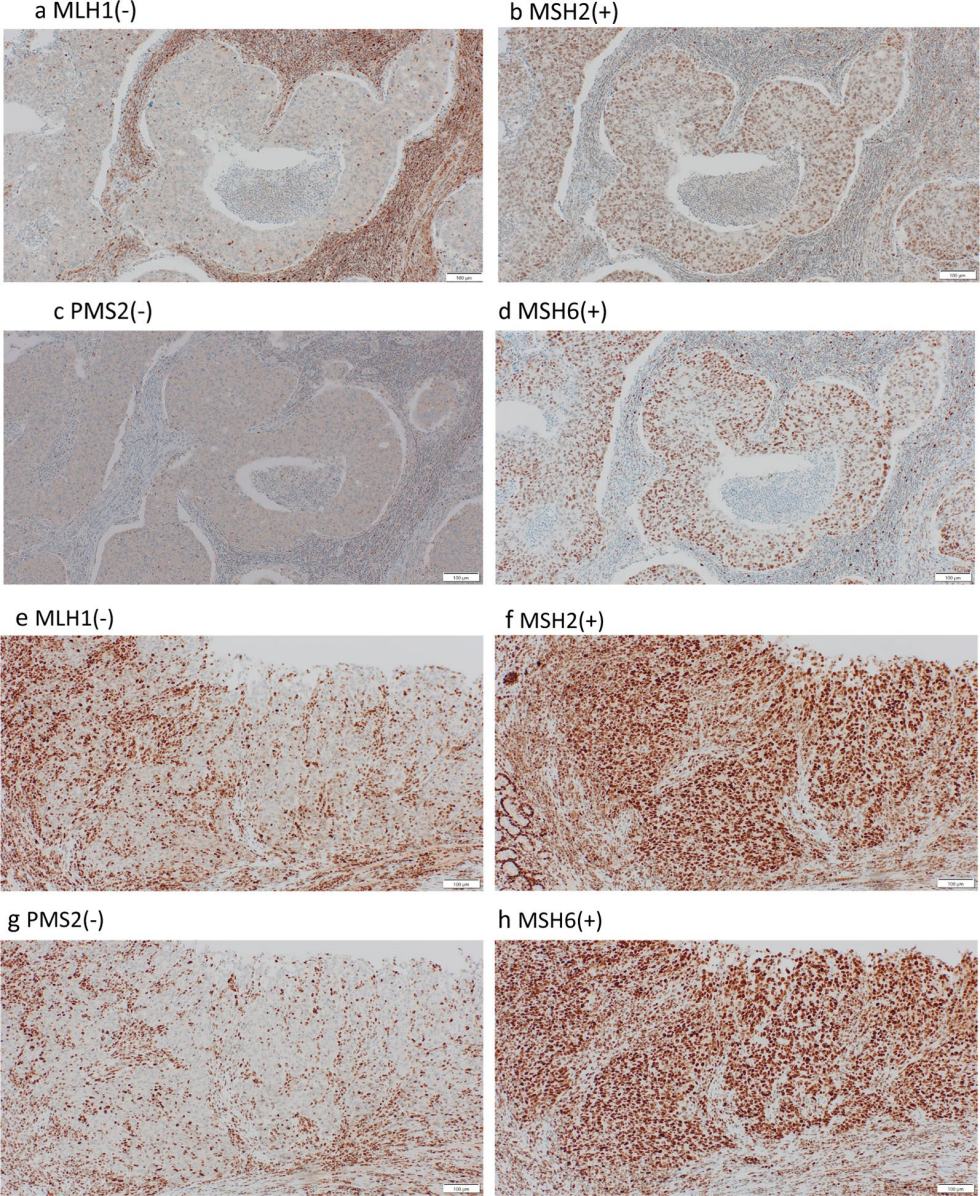
Immunohistochemical examination of mismatch repair proteins. The suprapancreatic lymph node (**a**–**d**) and stomach lesion (**e**–**h**) showed a lack of MLH1 (**a**, **e**) and PMS2 (**c**, **g**) expression in the tumor cell nuclei, in contrast to the positive staining for MSH2 (**b**, **f**) and MSH6 (**d**, **h**). These results indicated that both tumors were MSI-high. MSI, microsatellite instability

Subsequently, CT scans were performed annually, but no abnormal findings were observed. Nine and a half years after the lymph node excision, gastroscopy revealed a mixed raised and depressed lesion (0-IIa + IIc, according to the Japanese Classification of Gastric Carcinoma, 15th Edition [2]) at the angle of the previous ulcer scar (Fig. 3a). The patient had not undergone gastroscopy since the LNM excision. The pathological diagnosis, based on a biopsy of the 0-IIa + IIc lesion, was poorly differentiated adenocarcinoma. PET showed accumulation of F-18-FDG only in the main lesion in the stomach (Fig. 3b). As the relationship to previously resected lymph nodes was unknown, the clinical stage was estimated as cT1bN0M0 cStage IA (Japanese Classification of Gastric Carcinoma, 15th Edition [2]). Robot-assisted laparoscopic distal gastrectomy with Billroth-II reconstruction was performed; lymph node dissection was added to the D1 + level except for the No. 8a area (along the common hepatic artery), following gastric cancer treatment guidelines. No. 8a LN had already been dissected in the previous surgery and was difficult to detach due to severe adhesions. Macroscopic examination of the resected specimen revealed a 70 × 50-mm 0-IIa + IIc tumor extending from the lesser curvature to the posterior wall of the lower body (Fig. 3c). Histological examination showed clusters of atypical cells proliferating from the alveolar to the solid layers. Individual cells showed enlarged nuclei with polygonal and clear nucleoli. Lymphocytes densely infiltrated the tumor tissue, and lymphoid follicles formed in the periphery (Fig. 2b). The Epstein–Barr virus-encoded small RNA–in situ hybridization results were negative. This feature was similar to that of the previously removed suprapancreatic marginal lymph node. Both findings correspond to gastric carcinoma with lymphoid stroma according to the World Health Organization classification, 5th edition [3]. Immunohistochemical examination of mismatch repair (MMR) proteins showed a loss of MLH1/PMS2 expression, and retention of MSH2/MSH6 expression in both specimens (Fig. 4). Furthermore, both tumor was p53 wild type, CK7-positive, CK20-positive, CDX2-negative, and HNF4-alpha-positive. Based on these results, both gastric and lymph node lesions were diagnosed as MSI-high tumors. The final diagnosis was the gastric adenocarcinoma, suspected with lymph node metastasis, pT1bN1M0, pStageIB. The patient is alive and recurrence-free 2 years after gastrectomy.

## Discussion

We considered this case to be occult gastric cancer with LNM to the superior pancreatic margins. Although we ruled out cancer by endoscopic biopsy, the lesion that we believed to be an ulcer scar was most likely a cancer covered with regenerated epithelium. The extremely slow growth rate of this cancer may have resulted in the early cancer diagnosis 10 years later. We followed up annually with CT but did not perform gastroscopy, trusting the biopsy results. If gastroscopy had been performed considering the prognostic significance of the suprapancreatic lymph nodes in gastric cancer [4, 5], the cancer may have been diagnosed sooner. However, given the depth of the resected gastric carcinoma, the tumor grew extremely slowly, despite the presence of bulky LNM. Furthermore, it is already well known that *H. pylori* infection is an important factor in the development of gastric ulcer and gastric cancer [6]. Since a gastric ulcer scar was noted, the patient should have been tested for *H. pylori* and underwent periodic endoscopy and biopsy. It may have been too early to exclude the diagnosis of gastric cancer and follow-up only with CT.

Suprapancreatic lymph nodes, such as those found in this metastatic case, are known as extraperigastric nodes [7]. When a metastatic lymph node is discovered in an extraperigastric area without perigastric involvement, we refer to it as skip metastasis. The mechanism behind skip metastasis remains elusive. However, we do know that there exists a complex lymphatic flow from the stomach to the suprapancreatic area [8], making skip metastases a plausible occurrence. If the lesion remained at an early stage even 10 years after lymph node removal, it is exceptionally rare and valuable.

Common genetic mutations must be confirmed to conclude that two lesions resected at widely different periods have the same origin. In this case, genetic mutations were not examined; however, the histology of the two lesions was similar and characteristic of adenocarcinoma with lymphocytic infiltration. In addition, both specimens were presumed to be MSI-high based on the immunohistochemical examinations. These results strongly suggested that they had the same origin. Although histology did not prove malignancy and no other metastatic lesions were found, different origins would be rather unusual, because the same region of the stomach was affected.

Several cases of spontaneous regression of MMR-deficient colon cancer have been reported [9, 10]. However, spontaneous regression of gastric cancer is rare [11], and no reports of MSI-high gastric cancer were found in PubMed. Nonetheless, given that MSI-high gastric cancer has been reported to have a good prognosis [12, 13], it is possible that the primary lesion almost disappeared spontaneously and then remanifested after 10 years. In recent years, various biomarker molecules, such as human epidermal growth factor receptor 2 (HER2) and programmed cell death ligand 1 (PD-L1), have been discovered in addition to MSI, and molecular targeted therapies against them have been introduced into clinical practice [14–16]; however, in gastric cancer, the frequency of expression of these biomarkers is often low. In the present case, the two tumors, which were diagnosed at very different times, were inferred to be of the same origin because MSI-high tumors are rare; nonetheless, proving the molecular biological identity of the two tumors is difficult. The future discovery of various biomarkers is expected to help further elucidate the relationship between these tumors.

## Conclusion

This novel case is significant, because there have been no previous reports of gastric cancer with MSI-high characteristics in which the primary tumor was identified a long time after resection of metastatic lesions. With the rise of immune checkpoint inhibitors, the clinical characterization of MSI-high gastric cancers is expected to lead to advancements in their treatment.

## Data Availability

The data supporting the conclusions of this article are included within the article.

## Abbreviations

CA: Carbohydrate antigen
CECT: Contrast-enhanced abdominal computed tomography
F-18-FDG: Fluorine-18-fluorodeoxyglucose
GC: Gastric cancer
LNM: Lymph node metastasis
MMR: Mismatch repair
MSI: Microsatellite instability
PET: Positron emission tomography
